# Chronic Inflammation: Synergistic Interactions of Recruiting Macrophages (TAMs) and Eosinophils (Eos) with Host Mast Cells (MCs) and Tumorigenesis in CALTs. M-CSF, Suitable Biomarker for Cancer Diagnosis!

**DOI:** 10.3390/cancers6010297

**Published:** 2014-01-27

**Authors:** Mahin Khatami

**Affiliations:** Inflammation and Cancer Biology, National Cancer Institute (Ret), the National Institutes of Health, Bethesda, MD 20817, USA; E-Mail: mkgoodness@aol.com

**Keywords:** Yin and Yang of acute inflammation, immune surveillance, conjunctival associated lymphoid tissues (CALTS), mast cells, tumor-associated macrophages (TAMs), cancer biomarkers, targeted therapies, neurodegenerative and autoimmune diseases

## Abstract

Ongoing debates, misunderstandings and controversies on the role of inflammation in cancer have been extremely costly for taxpayers and cancer patients for over four decades. A reason for repeated failed clinical trials (90% ± 5 failure rates) is heavy investment on numerous genetic mutations (molecular false-flags) in the chaotic molecular landscape of site-specific cancers which are used for “targeted” therapies or “personalized” medicine. Recently, unresolved/chronic inflammation was defined as loss of balance between two tightly regulated and biologically opposing arms of acute inflammation (“Yin”–“Yang” or immune surveillance). Chronic inflammation could differentially erode architectural integrities in host immune-privileged or immune-responsive tissues as a common denominator in initiation and progression of nearly all age-associated neurodegenerative and autoimmune diseases and/or cancer. Analyses of data on our “accidental” discoveries in 1980s on models of acute and chronic inflammatory diseases in conjunctival-associated lymphoid tissues (CALTs) demonstrated at least three stages of interactions between resident (host) and recruited immune cells: (a), acute phase; activation of mast cells (MCs), IgE Abs, histamine and prostaglandin synthesis; (b), intermediate phase; down-regulation phenomenon, exhausted/degranulated MCs, heavy eosinophils (Eos) infiltrations into epithelia and goblet cells (GCs), tissue hypertrophy and neovascularization; and (c), chronic phase; induction of lymphoid hyperplasia, activated macrophages (Mϕs), increased (irregular size) B and plasma cells, loss of integrity of lymphoid tissue capsular membrane, presence of histiocytes, follicular and germinal center formation, increased ratios of local IgG1/IgG2, epithelial thickening (growth) and/or thinning (necrosis) and angiogenesis. Results are suggestive of first evidence for direct association between inflammation and identifiable phases of immune dysfunction in the direction of tumorigenesis. Activated MFs (TAMs or M2) and Eos that are recruited by tissues (e.g., conjunctiva or perhaps lung airways) whose principal resident immune cells are MCs and lymphocytes are suggested to play crucial synergistic roles in enhancing growth promoting capacities of host toward tumorigenesis. Under oxidative stress, M-CSF may produce signals that are cumulative/synergistic with host mediators (e.g., low levels of histamine), facilitating tumor-directed expression of decoy receptors and immune suppressive factors (e.g., dTNFR, IL-5, IL-10, TGF-β, PGE2). M-CSF, possessing superior sensitivity and specificity, compared with conventional markers (e.g., CA-125, CA-19-9) is potentially a suitable biomarker for cancer diagnosis and technology development. Systematic monitoring of interactions between resident and recruited cells should provide key information not only about early events in loss of immune surveillance, but it would help making informed decisions for balancing the inherent tumoricidal (Yin) and tumorigenic (Yang) properties of immune system and effective preventive and therapeutic approaches and accurate risk assessment toward improvement of public health.

## 1. Introduction

During the evolutionary process, inflammation became known as an inherent self-limiting property of the immune system to protect the body against harmful or foreign agents (stimuli or irritants). Historically, in the 19th century Rudolph Virchow noted that the signs of inflammation were four-“*redness, and swelling with heat and pain*”. In 1909, observations by Ehrlich that tumor cells are recognized and eliminated by the immune cells were later evolved to the theory of immune surveillance (cancer surveillance), an effective property of immunity to monitor/survey organ systems and destroy those internal or external elements that are useless or threaten the body’s survival [1,2,3]. Since these historical observations a role for inflammation in the genesis and progression of many acute diseases (e.g., sepsis, pneumonia, meningitis or major trauma), allergies (e.g., asthma, emphysema, skin and ocular inflammatory diseases), a wide range of age-associated chronic illnesses, neurodegenerative and autoimmune diseases (e.g., rheumatoid arthritis, atherosclerosis, dementia, Alzheimer’s, multiple sclerosis, hypertension, diabetes, stroke and cardiovascular complications, colitis, gastritis, hepatitis, nephritis, prostatis, pancreatitis, appendicitis, thyroiditis, opthalmitis, Grave’s disease, fibromyalgia, Bechet’s, esophagitis, neuritis, lupus, Parkinson’s, psoriasis) and many cancers (e.g., lung, colon/rectal, breast, cervical, prostate, bladder, liver, gall bladder, appendix, ovarian, thyroid, pancreas, brain, hematologic malignancies) has been reported in the literature [3,4,5,6,7,8,9,10,11,12,13,14,15,16,17,18,19,20,21,22,23,24,25,26,27,28,29,30,31,32,33,34,35,36,37,38,39,40,41,42,43,44,45,46,47,48,49,50,51,52,53,54,55]. However, with regard to cancer, due to tremendous resistance of the politicians in the decision making roles in cancer community, the crucial impact of the immune surveillance or inflammation in tumor growth was practically forgotten and ignored in the past century [3,56].

The evidence for ignoring and downplaying the essential role of immune surveillance in cancer prevention and/or treatment comes from the fact that except for our “accidental” discoveries that were established in 1980s on experimental models of acute and chronic inflammatory diseases that resulted in tumorigenesis, little/no data is available to demonstrate:

(a) A direct evidence for an association between inflammation-induced immune alterations that would lead to tumorigenesis and angiogenesis;
(b) Identification of cellular composition of host/target tissue resident or recruited immune and non-immune cells and the nature of interactions between mediators, under a wide range of stimuli-induced immune response profiles that would result in cellular growth and site-specific cancers;
(c) Time-course kinetics of developmental phases of immune response alterations that are identified during early stages of tumorigenesis and angiogenesis which potentially are preventable, reversible or correctable.

Furthermore, the ongoing debates, misunderstanding and controversies on the role of inflammation in cancer have been extremely costly to taxpayers and cancer patients due to molecular false-flagging of mediators in site-specific cancers and repeated failed therapeutic approaches and clinical trials (see details in Section 9) [3,56].

Even in the past few years that the cancer community began to appreciate the roles that immune/inflammatory processes play in multi-step carcinogenesis and therapeutic approaches, little serious efforts are directed toward comprehending the early immune dynamic alterations that occur in susceptible tissues that result in the progression of immune dysfunction and destruction of tissue architectural integrity and function and carcinogenesis, particularly during the aging process as most of the site-specific cancers occur in older adults. Furthermore, there are tremendous knowledge gaps on the composition of host resident immune and non-immune cells and/or the interactions with recruited cells in the disease outcomes. In addition, little is known about how biology of aging together with sustained oxidative stress could disrupt the orderly biological, physical and/or mechanical integrity of the target tissues that allow cancerous cells to take over the machinery of cellular components, including the immune system for their enhanced growth requirements. The impact of chronic inflammation in the disruption of cellular compartments [e.g., extracellular/intracellular matrix interactions, autophagy and lysosomal or Golgi apparatus (protein anabolic/catabolic balances, clearance and/or accumulation of abnormal protein folding), oxidative damage to mitochondria and altered oxido-redox ratios in changing the energy or metabolic status of host] in the multistep carcinogenesis, angiogenesis and metastasis are important topics in cancer biology that are not fully understood [3,26,39,40,44,46,56].

This perspective focuses primarily on overview of the recent definitions and hypotheses on the role of acute and chronic inflammation and the function of immune surveillance in health and diseases, with emphasis in cancer. Analyses of a series of data from our earlier discoveries on inflammation-induced systematic alterations of immune dynamics and induction of massive hyperplasia of conjunctival-associated lymphoid tissues (CALTs) will be summarized. It is suggested that the extent of interactions and synergies between activated resident (local, host) immune cells and those that are recruited (e.g., Eos, TAMs) to the site of injury will determine the outcomes of host responses. Outlines of an NCI-invention on standardizing cancer biomarkers criteria (data elements) as a foundation of a database using M-CSF as a potentially suitable marker for cancer diagnosis, validation and technology development will be presented.

## 2. Acute Inflammation: Protective, Self-Terminating Property of Immune Surveillance

Effective immunity that is beneficial to health is provided through natural pleiotropy or duality (polarity) of the immune system via self-terminating properties of acute inflammation (immune surveillance). In brief, acute inflammation provides the organ systems the ability to return to normal physiological function after encountering and defeating internal or external useless and/or harmful/foreign elements, so that the body could survive and thrive throughout life. Tissue exposure to foreign elements (stimuli) including infective agents, microorganisms (microbiota) or pathogens (e.g., viruses, bacteria, parasites), allergens, carcinogens/toxins, biological, chemical or environmental hazards (e.g., pollen, dust, termites, over the counter drugs, asbestos, paints, detergents, hair sprays, cosmetics, food additives, pesticides and genetically modified foods), oxidized metabolites (e.g., crystalline uric acid), non-functional or defective proteins/enzymes, defective or mutated genes, DNA/RNA, hypo-, hyper-methylated epigenetic molecules, useless and/or defective cells (e.g., polyclonal B cell complexes, senescent and cancerous cells) would induce specific local and/or systemic signals for appropriate immune responses [3,4,9,11,12,13,14,38,39,40,50,51,52,53,54,55,56,57,58,59,60,61,62,63,64,65,66].

**Figure 1:**
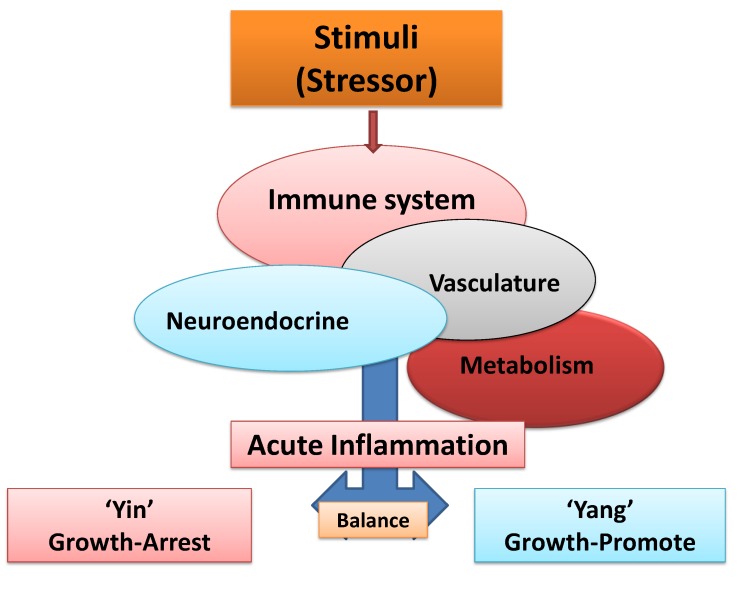
Schematic representation of balance between “Yin” (growth-arresting) and “Yang” (growth-promoting) of acute inflammation. Inflammatory processes involve elaborate, precise and interdependent cross-talk between immune and non-immune systems (e.g., vasculature, neuroendocrine and metabolic pathways) to combat and destroy foreign elements and injured host tissue and to neutralize, resolve and terminate inflammation and repair and reconstruct the damaged tissues.

**Figure 2:**
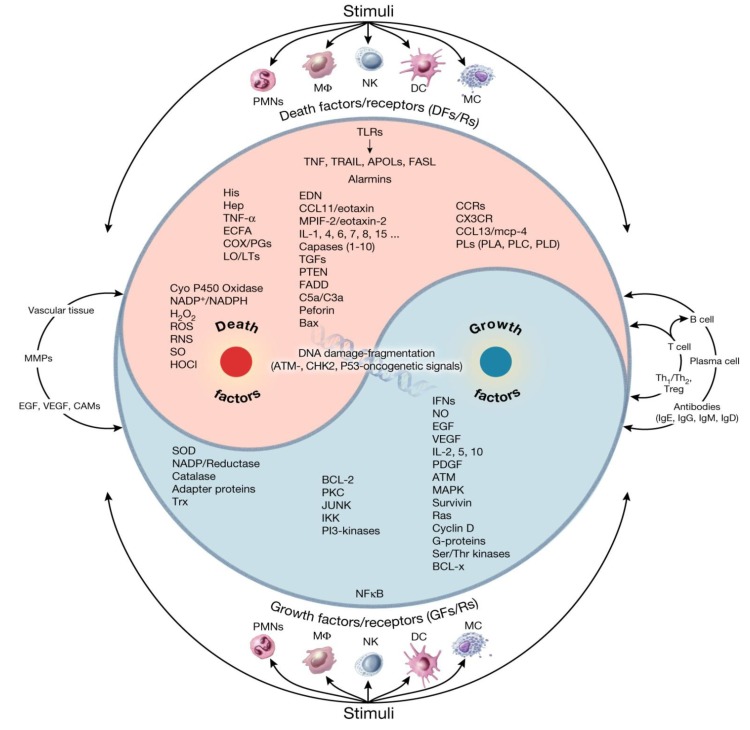
Schematic representation of Yin (Death factors) and Yang (Growth factors) processes in acute inflammation. Reproduced from Khatami [38] with permission from Informa Healthcare, all rights reserved.

Recently, acute inflammation was defined as the balance between two highly regulated and biologically opposing arms of the immune system, termed “Yin” (apoptosis, initiating, growth-arresting, pro-inflammatory or tumoricidal) and “Yang” (wound healing, terminating, growth-promoting, anti-inflammatory or tumorigenic) responses that are facilitated by intimate responses from non-immune systems (e.g., vasculature, neuroendocrine and metabolic pathways) (Figure 1 and Figure 2) [3,38].

Briefly, tissue stimulation induces cell mediated and/or humoral immunity (CMI, HI) via a highly sophisticated and precise communication between activated innate [e.g., natural killer cells (NKs), macrophages (MFs), dendritic cells (DCs), mast cells (MCs)] and/or adaptive/effector immune cells [e.g., T and B cells, and subpopulations (e.g., cytotoxic T cells, Th1, Th2, Treg)] as well as, contributions of signals from the activated vasculature, neuroendocrine and metabolic pathways with the mission to:

(a) Encounter, sense/recognize, destroy/defeat and eliminate foreign elements and the injured host tissue during “Yin” (tumoricidal) responses;
(b) b) Terminate, neutralize or resolve inflammatory responses and repair or remodel the host tissue during “Yang” (tumorigenic) responses.

The major outcomes of an acute inflammation are lymphocyte-derived clonal expansion, increased synthesis of allergen-, or pathogen-specific antibodies and synthesis of plasma and memory T and B cells, so that when the body is exposed to the same stimuli, the immune system is prepared to unleash appropriate responses toward hazardous material [3,38,39,40

## 3. Specialized, Shared and Synergistic Features of APCs

In general, during cell mediated immunity (CMI), antigen presenting cells (APCs) are responsible for recognition, uptake and clearance of a wide variety of external or internal foreign elements (stimuli). The host defense system also has the capability for tolerance and remembrance through regulatory T (Treg) or memory T and B cells. CMI that are mediated through MFs and DCs are generally specialized for combating viruses and bacteria. CMI that are mediated through NKs and/or cytotoxic T cells (CTs) play crucial roles in the elimination of internal microorganisms (virus-infected cells and neoplastic or cancerous cells). Under certain inflammatory conditions or perhaps in certain peripheral tissues [e.g., skin, lung airways, conjunctival-associated lymphoid tissues (CALTs) or gut-associated lymphoid tissues (GALTs)] reverse roles in immune responses occur when B cells, within adaptive immunity, act as APCs while MCs, within innate immune cells, become effector cells. In such occasions B cells are responsible for sensing, editing and processing specific microorganisms or allergens/antigens. Activated B cells become transformed to plasma cells and synthesize specific antibodies (e.g., IgGs, IgM, IgD, IgA, IgE) and appropriately sensitize or activate other immune cells. In such cases, HI and expression of specific antibodies seem to principally determine which innate immune cells are required for further response processes and for appropriate induction of memory B and T cells [3,13,14,27,28,30,35,37,39,44,47,49,50,51,52,53,54,55,56,57,58,59,60,61,62,63,64,65,66,67,68,69,70,71,72,73,74,75,76,77,78].

Therefore, it is reasonable to hypothesize that the combined interdependent cellular and molecular factors including the type and susceptibility of host resident (local) immune and non-immune cell composition, as well as the nature and potency, duration and route of exposure to stimuli are principal confounding determinants in the outcomes of acute or chronic responses. For example, in tissues such as the CALTs or perhaps lung airways and GALTs, B and plasma cells are the first line of defense when exposed to stimuli to express specific IgE antibodies for binding and sensitizing MC-IgE-fcε (epsilon) receptor molecules for appropriate responses (e.g., degranulation and release of pre-formed or newly synthesized mediators) during acute hypersensitivity or anaphylactic reactions (see details in Section 8) [3,35,37,59,60,61,62,63,64,65].

## 4. Unresolved Inflammation: Common Denominator in Induction of Age-Associated Chronic Diseases or Carcinogenesis

**Figure 3:**
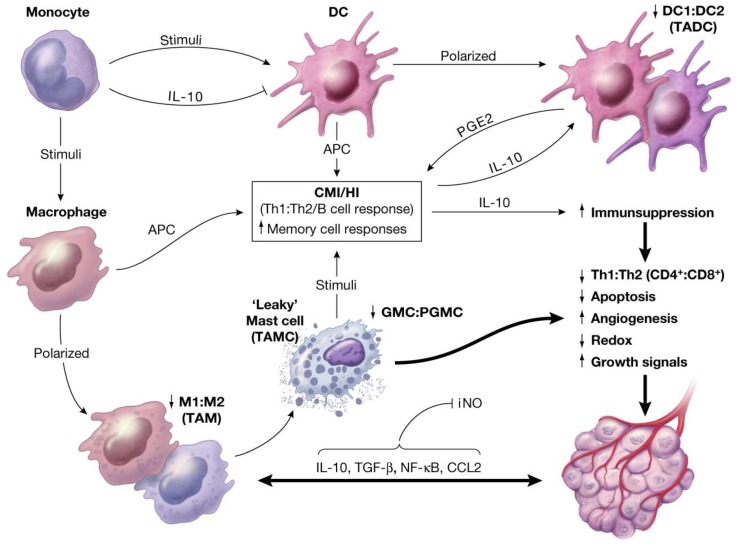
Schematic representation of oxidative stress-induced changes in the balance between inherent polarity (duality, Yin and Yang) of immune responses and the induction of immune suppression in immune-responsive tissues toward tumorigenesis and angiogenesis. Reproduced from Khatami [38] with permission from Informa Healthcare, all rights reserved.

Unresolved inflammation was hypothesized as the loss of balance between Yin and Yang of acute inflammation [3,38,39,40]. Under a variety of inflammatory conditions, the two opposing arms of Yin and Yang responses, in an attempt to resolve inflammation, would influence differentiation and polarization of self, or other immune cells into subpopulations [e.g., transformation of immature DC1(tumoricidal) to mature DC2 (tumorigenic, TADCs); phagocytic and tumoricidal MFs (M1) to tumorigenic form (M2 or TAM), or granulated MCs (tumoricidal) transformation to partially granulated “leaky” (LMCs or tumor associated-TAMCs)]. As schematically represented in Figure 3, chronic or unresolved (long-standing, oxidative stress, sub-clinical or persistent) inflammation could change the dynamics of immune responses through exaggerated and/or mismatched co-expression of apoptotic and growth factors in target tissues, creating an “immunological chaos” and causing minor or major damages in the structure and function of susceptible target tissues. These response changes could lead to initiation and manifestation of a wide range of neurodegenerative and autoimmune diseases, or cell growth promotion, polyps or benign tumors, pre-cancer or neoplasia, hyperplasia, tumor growth and/or site-specific cancers, angiogenesis and metastasis [3,38,39,40,56].

**Figure 4:**
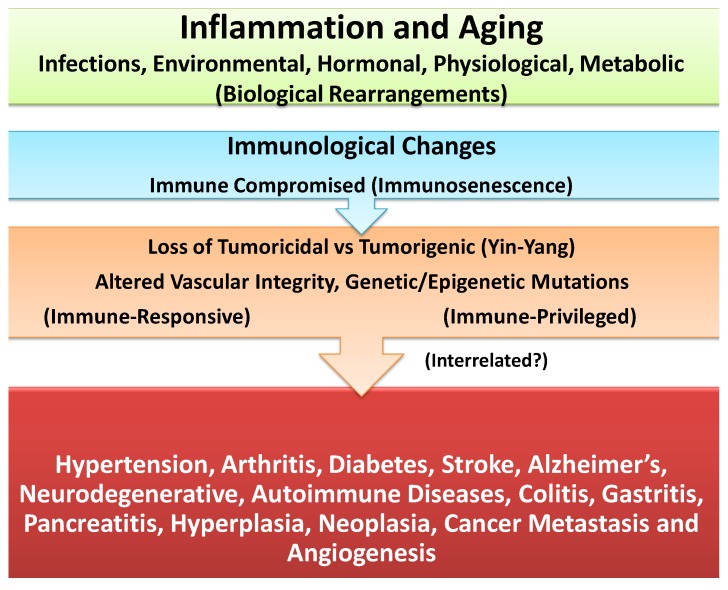
Schematic representation that longevity and oxidative stress are co-morbidity and co-mortality risk factors in the induction of biological and immunological senescence that are involved in instabilities of cellular and genetic/epigenetic and vascular functions. The altered cellular functions differentially impact immune-privileged and immune-responsive target tissues and lay a foundation for initiation, progression and manifestation of a wide range of age-associated chronic diseases and/or site-specific cancers that are potentially interrelated.

In addition, longevity-associated with alterations in the availability, expression levels, and/or transport capacities of important hormones, metabolites/solutes and/or other biological components (e.g., receptor, adaptor or surface molecules, reducing or oxidizing enzymes, repair or transporter proteins) could cause minor or major defects in cellular components [e.g., mitochondrial oxidative metabolism (mitophagy), protein anabolic-catabolic balance (autophagy) or lysosomal defects in protein recycling or clearance and/or accumulation of useless oxidized cells, proteins, metabolites, or lipids complexes, changes in nuclear components affecting genetic/epigenetic structure or function]. The age-associated biological changes lead to biological rearrangements/compensations (biological senescence) in organ systems. Furthermore, age-induced minor or major alterations in innate and adaptive immune cell responses (immunosenescence) could further enhance the risks for initiation and progression of diverse chronic conditions or cancer (Figure 3 and Figure 4) [3,38,39].

Cancerous cells should be regarded as defective cellular byproducts whose oncogenic growth features are normally and routinely monitored and arrested by the body’s effective immune surveillance as any other pathogens, chemical, environmental or biological hazards. As discussed above, oxidative stress and longevity which are considered as co-morbidity and co-mortality risk factors could alter the functions of cell/organ systems (e.g., oxidative metabolism, autophagy, vasculature toning, DNA/RNA and/or hypo-, hyper-methylation of epigenetic components) and contribute to the retardation and ineffectiveness of immune response profiles. Therefore, loss of effectiveness in immune surveillance could induce expression of response mismatch or immunological chaos (immune tsunami) significant enough to cause response shifts and erosion of the architectural integrity and function of tissue leading to initiation and progression of nearly all age-associated chronic diseases or many site-specific cancers (Figure 4) [3,38,39,56].

## 5. Differential Impact of Inflammation in Immune-Responsive and Immune-Privileged Tissues and Chronic Diseases or Cancer

Recently, it was hypothesized that inflammation differentially impacts the tissues that are immune-responsive (e.g., epithelium, endothelium, fibroblasts, mucus-secreting goblet cells, stroma, vasculature, lymphoid organs) or immune-privileged (e.g., central nervous system/CNS, blood brain barrier/BBB, avascular cornea, optic nerve, neuroretina, reproductive organs) as the basis for initiation, progression and manifestation of a wide range of autoimmune and neurodegenerative diseases or cellular growth and carcinogenesis, metastasis and angiogenesis (Figure 4) [3,39,40,56].

Other subcategories of host tissues that inflammatory responses could influence the disease outcomes include the insulin-sensitive/dependent tissues (e.g., muscle, adipocytes or liver cells) and insulin-insensitive/independent tissues (e.g., vasculature, endothelial or neuronal tissues) for glucose transport or metabolism. Hyperglycemia of diabetes (glucose toxicity) or insulin resistance is likely to differentially influence these tissues during the induction of diabetes and cardiovascular complications, stroke or neuronal dysfunction (e.g., neuropathy, retinopathy, nephropathy) and causes increase or decrease risks for site-specific cancers [3,39,40].

## 6. Interactions and Synergies between Resident and Recruited APCs in Host Tissue

Stimuli-induced inflammatory cross talks between resident (host) and recruited inflammatory cells are complex and not fully understood. Review of a large amount of data on immune response profiles in target tissues suggests that polarization of immune cells is accompanied by expression of mediators whose effects are different from their non-polarized phenotypes (resting status) in order to act as tumor suppressors (growth-arresting), or tumor promoters (growth-promoting) [3,39,40,43,44,47,50,56,57,58]. Factors with known dual functions include TLRs, MCP-1-CCL2, M-CSF, TGF-β, GM-CSF, histamine, heparin, TNF-α-TNFR, VEGF, CAMs, MMPs, prostaglandins, surface antigens, adaptor molecules or cell recognition molecules (e.g., CD2, CD11, CD18, CD22, CD25, CD 50, CD54, CD63, CD69, CD88), cytokine suppressor molecules (e.g., S100 family of calcium-binding proteins), enzymes (e.g., tryptase/chymase, neutrophil-derived serine proteases, indolamine 2,3-dioxygenase, lipases or membrane metalloproteases/MMPs), peroxynitrite, cytokines/chemokines, interleukins (e.g., CCL2, CXC, Il-2, IL-3, IL-5, IL-10, IL-12, IL-13) or interferons (e.g., IFN-γ), ECFA, SCF, c-kit, antibodies (e.g., IgE, IgG isotypes, IgA, IgM), platelet-derived growth factor (PDGF) as well as expression products of gene activation pathways (e.g., p53, p27, p70, MAPKs, KRAS, BRAF, ALK, Myc, BCR, ABL, MGMT, TKIs, PI3ks) from mutated DNA, hypo-hyper-methylation products that are reported in chronic diseases or cancer [3,4,5,6,7,8,9,10,12,16,17,18,19,20,27,28,29,30,31,32,33,34,35,36,37,38,39,40,41,42,43,44,45,46,47,48,49,50,51,52,53,54,55,56,57,58,59,60,61,62,63,64,65,66,67,68,69,70,71,72,73,74,75,76,77,78,79,80].

## 7. Circumstantial Evidence for Association between Inflammation and Cancer

Circumstantial evidence for a link between inflammation and cancer has been documented for many decades (Table 1) [3,37,38,39,40,44,56]. However, the cancer community has only begun to accept and appreciate the importance and significance of inflammation in cancer biology in the last decade [3,40,56]. It is now generally accepted that chronic inflammation is a precancerous state of cells; and that the process of carcinogenesis is initiated and facilitated by unresolved inflammation. Furthermore, in the last few years increasing numbers of funded projects have focused on the role of numerous inflammatory mediators relating to basic and clinical cancer research, immunotherapeutic approaches and related technologies. These studies include reports on the dual properties of immune cell responses and their involvement in site-specific cancers [3,8,15,18,19,20,22,23,28,37,38,39,40,43,44,47,56,57,58,67,77,78,79,80,81,82,83,84,85,86,87].

**Table 1:** Growing list of reported data on clinical and epidemiological findings for circumstantial evidence on association between chronic prior injuries and site-specific cancers.

Tumor (Cancer) Site	Inflammatory Disease
Bowel/Colon	Ulcerative Colitis (Crohn’s)
Urinary Bladder	Schistomiasis, Stones, Catheters
Prostate	Prostatitis---PIA-PIN
Breast	Inflammatory Conditions
Cervical	Pericarditis?
Esophagus	Barretts’
Pancreas	Pancreatitis
Stomach	Gastric Infection (e.g., H Pylori)
	Ulcers/Gastritis
Lung	Asthma, Emphysema, Smoking
Liver	Hepatitis B, C
Thyroid	Thyroiditis
Conjunctiva	Conjunctivitis/CALTs (?)
Skin	Melanomas Burns/Radiations (?)
Uveal	Melanomas Uveitis (?)
Others	Cystitis (?)
	Hyperplasia of GALTs (?)

## 8. Direct Association between Inflammation and Tumorigenesis and Angiogenesis: Interactions between Resident and Recruited APCs in Experimental Models of Acute and Chronic Ocular Inflammatory Diseases

To date, the time-course kinetics of inflammation-induced alterations in immune dynamics that were reported in 1980s on models of acute and chronic ocular inflammatory diseases are perhaps the best and only evidence for sequential interactions between activated resident and recruited APCs that resulted in tumorigenesis and angiogenesis in CALTs [3,35,36,37,39,45,77,87,88,89,90]. Analyses of data will be reexamined below to demonstrate the importance of synergistic interactions between host immune cells and those cells that are recruited during the progressive alterations of immune responses in the induction of tumorigenesis.

**Figure 5:**
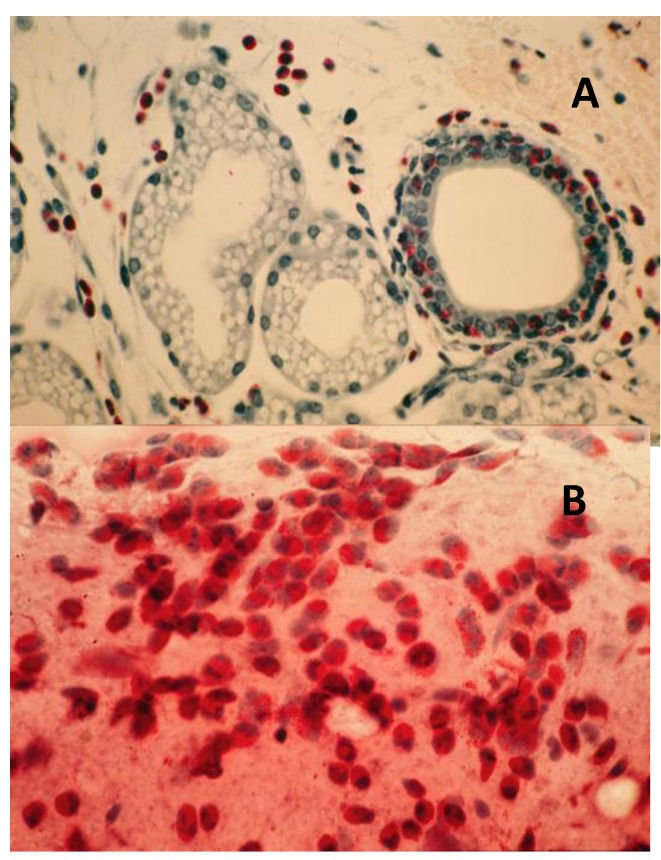
Induction of down regulation in CALTs and eosinophil infiltration. Panel A; heavy eosinophils infiltration into GCs. Panel B; heavy eosinophils infiltration in conjunctival secretions from animals that were repeatedly challenged with topical application of FLOA and systemically immunized with A. Suum. Reproduced from Khatami *et al*. [35], @1984 American Medical Association, all rights reserved.
(d) **Induction of tumorigenesis with mixture of antigen and TPAs:** Animals that were topically treated with a mixture of FLOA and TPAs developed tumor-like lesions within 6 months after commencement of sensitization. These preliminary observations were suggestive of additive impact of TPAs that shifted the kinetics of altered immune responses and tumorigenesis to earlier events, perhaps through activation of protein kinase C (PKC) and/or other tumor growth pathways [3,36,37].
(e) **Antibody profiles (humoral immunity, HI):** Repeated stimulation of tissues and the induction of tumorigenesis produced significant increase in the expression of immunoglobulin isotypes (e.g., IgG1/IgG2 ratios) in culture media of massive hyperplastic CALTs, suggesting that frequent exposure to large dosage of antigen into substantia propria, or sub-epithelial tissues altered antibody profiles [3,37,88]. Indirect support for these observations came from the studies when guinea pigs were injected sub-conjunctivally with low dosage (or less frequent exposure) of nematode *Onchocerca lienalis* microfilaria; where no significant changes in biosynthesis of local IgG1 to IgG2 antibodies were observed in the cultures [3,90]. Others demonstrated diversities in the expression of cytokines and antibodies in B lymphocytes in humans and transgenic CCL2-deficient mouse models in the induction of inflammatory diseases or carcinogenesis [27,32,37]. The B lymphocytes in CCL2-deficient mice were shown to be unable to synthesize normal profiles of subclasses of antibodies, and continued synthesis of high levels of IgG2a and IgG2b, and low levels of IgG1, after immunization [3,27,28,32,37].

**Figure 6:**
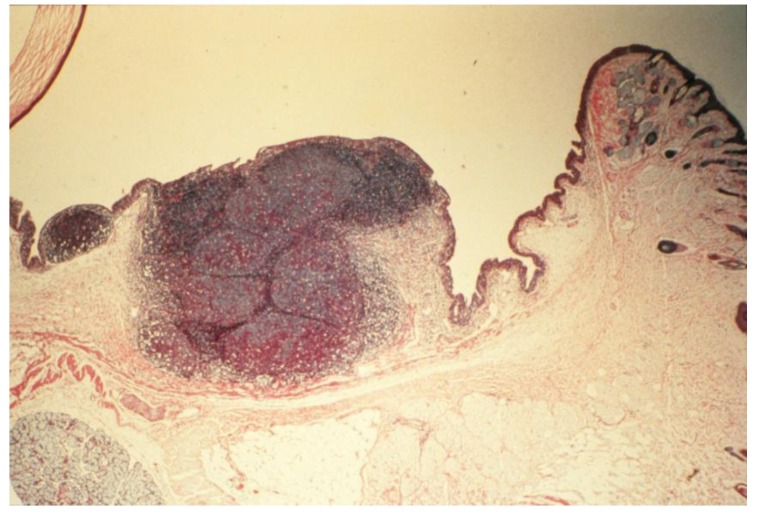
Histopathologic section of eyelid of repeatedly topically immunized and challenged guinea pig showing massive hyperplasia of CALTs. Reproduced from Khatami *et al.* [36], @1989 American Medical Association, all rights reserved.
(f) **Statistics and data analyses:** From a total of 400 eyes that were examined, 12/40 (30%) of the eyes from animals that were not sacrificed during earlier immunization periods developed tumor-like lesions or hyperplasia of CALTs. These preliminary data that suggested that tumor developed primarily in animals that initially produced minimal early type 1 hypersensitivity reactions, deserve further investigations [3,36,37].

Acute and chronic ocular inflammatory diseases were induced in conjunctival-associated lymphoid tissues (CALTs) in guinea pig eyes by topical (unilateral and/or bilateral) application of fluoresceinyl-ovalbumin (FLOA, antigen) in the presence or absence of infective agents (e.g., Ascaris Suum/A. Suum parasite or its extracts), adjuvant or tumor promoting agents (TPAs) for up to 30 months [3,35,36,37,45,87,88,89,90]. At least three distinct phases of immune response dysfunction were identified as outlined in the following:

(a) **Acute-phase response:** Initiated 9 days after topical sensitization and challenge with FLOA. Clinical and histopathological findings included a combination of strong or weak acute (type 1, hypersensitivity) reactions, tearing, scratching and conjunctival edema, milky secretions, IgE-dependent mast cells (MCs) degranulation, release of histamine and PGs, vascular hyperpermeability and coagulation of lipid-protein complex exudates. Time-course kinetics of release of histamine and PGs (6-keto-PGF-1α, a stable product of PGI2) into tears suggested that histamine (a potent and preformed vasoactive agent) was a primary mediator that activated membrane arachidonic acid metabolic pathways, e.g., activation of cyclooxygenase and lipooxygenase and the synthesis and release of prostanoids (PGs). No correlation was found between circulating homocytotropic-IgE and the degree of clinical reactions. Further studies with untreated eyes, or lung tissues and ocular challenges of new-born babies from sensitized animals suggested high affinity binding of IgE-MCs-Fc-ε receptors and removal of circulating IgE by MCs throughout the body [3,35,37,89].
(b) **Intermediate-phase responses; induction of down-regulation phenomenon:** Occurring within 2 months of continuous sensitization and challenge. Findings included minimal tearing or tissue edema, loss (exhaustion) of number of functional or tumoricidal (mature) MCs, extensive infiltration of eosinophils into subepithelium and mucus-secreting GCs, tissue hypertrophy and neovascularization (Figure 5). Topical application of compound 48/80, a mast cell degranulating agent, to desensitized eyes produced little reactions. In contrast, histamine applied to desensitized eyes produced strong type 1 reactions. These observations further demonstrated that mast cells were exhausted (non-functional) while histamine receptors were not affected by repeated challenges at this stage of sensitization [3,37,87].
(c) **Chronic-phase response, induction of tumorigenesis and angiogenesis:** Occurring between 12 to 30 months of repeated tissue stimulation with FLOA. Findings included induction of tumor-like lesions in conjunctival tissues, angiogenesis, massive lymphoid hyperplasia, follicular formation with germinal centers, activated MFs, presence of histiocytes, loss of lymphocyte capsular membrane and extension of various sized B lymphocytes into surrounding epithelial tissues, increased swollen GCs, increased degranulated or partially granulated (“leaky”) MCs, involvement of lymphatic channels, extensive epithelial thickening (growth) and/or thinning (necrosis) often noted in the same tissue sections. Cross-sectional areas of massive hyperplastic lymphoid nodules from animals that were continuously challenged with antigen were at least five times larger than lymphoid tissues in normal-untreated animals (Figure 6) [3,36,37,39,45].

The results of these studies are suggestive of the first evidence for a direct association between inflammation and tumorigenesis [3,37,56]. Analyses of data also led to a first report on time-course kinetics of identifiable early steps of inflammation-induced altered immune dynamics that would lead to tumorigenesis and angiogenesis [37]. These findings also suggest that the signals originated from exhausted or “leaky” MCs (e.g., low level release of histamine, independent of IgE-Fcε-receptor binding or perhaps “unscheduled” expression of cytokines/chemokines or enzymes from immature MCs) in CALTs are involved in recruiting other inflammatory cells such as Eos and/or MFs to express additional factors that are immune suppressive and could facilitate tumorigenesis [3]. The highly versatile roles of mast cells as effector cells in the induction of a wide range of acute and chronic inflammatory diseases including vernal conjunctivitis, delayed type hypersensitivity (DTH), asthma/emphysema and other allergic reactions in human or experimental models of hypersensitivity conditions and cancers have been extensively reported [3,12,13,14,35,37,53,57,59,60,61,62,63,64,65]. A number of MCs mediators with dual functions including increased and continuous release of histamine in tissue, serum or tear, as well as expression of IgE or IgG isotypes, immunoglobulin-free light chains (FLCs) and numerous other preformed or newly synthesized cytokines/chemokines and enzymes and their interactions with activated T and B cells in the induction of inflammatory diseases or cancer and therapeutic approaches have been documented [3,59,60,61,62,63,64,65,76,77,78,87,88]. Furthermore, review of immunopathological data on human and experimental models of conjunctivitis, conjunctival marginal zone B lymphoma or squamous cell carcinoma demonstrate a range of cellular and molecular reactions including appearance of yellowish cornea, yellow stringy exudates composed of mucus, deposition of amyloid in mucosal-associated lymphoid tissue, involvement of epithelial cells, eosinophils, eosinophil granules and mononuclear cells [3,32,59,60,61,62,63,64,65,67,69,70,71,73,74,76,77,78,87,88,89,90,91].

Further analyses of data on the interactions between MCs and T, B or plasma cells in the induction of inflammatory diseases or cancers, demonstrate that many of the isolated time points that are reported in the study outcomes, indirectly support the systematic time-course kinetics of inflammation-induced tumorigenesis and angiogenesis in CALTs that were established over 20 years ago. These studies are considered “accidental” discoveries since in 1980s our research team was not involved in cancer research and we had no idea of the importance or significance of these findings in cancer research until the author came to NCI/NIH in 1998. At NCI she was given the responsibility to review the cancer data and major clinical trials [e.g., prostate-lung-colorectal-ovarian (PLCO) Cancer Screening Trials] and to develop concepts for molecular diagnosis/biomarkers, prevention, and utilization of biorepositories (biospecimen) and design of cohort clinical studies. The results of these challenging efforts to promote the role of inflammation in cancer research are documented in NCI/NIH records and published articles [3,37,39,45,48,56,57,77]. 

Confirmation and extension of these studies and comparison of data with other host organs/tissues with similar (e.g., lung airway or GALTs) or different (e.g., liver, kidney or pancreas) immune and non-immune cell compositions seem to be important logical approaches for gaining insightful appreciation of the biology of cells/tissues and the roles that resident and recruited immune and/or non-immune cells play in the induction of chronic diseases and/or cancer.

## 9. Assessment of Cancer Claimed “Targeted” Therapies or “Personalized” Medicine: False Foundation and Failed Outcomes!

The ongoing debates, controversies and continued misinterpretation of isolated data on the role of inflammation, whether it prevents cancer or it causes cancer have been extremely costly for taxpayers and cancer patients when clinical trials and therapeutic approaches are decided [3,56]. Despite the heavy public investment (well over a trillion dollars from public and private sources spent in the last four decades alone) on war against cancer, data on cancer biology is still fragmentary, fuzzy and confusing and cancer remains a mystery that continues to bring death and misery to millions of growing populations around the globe. The claims for “progress” are principally based on identification and characterization of too many out-of-focus molecular entities, with extensive and disproportionate emphasis and funding of infinite number of genetic mutations and their expression products, projects that are proven worthless, but highly fashionable and fundable by the decision makers in the cancer community. The molecular entities that are reported in the landscape of site-specific cancers and labeled as genetic mutations or “mutational drivers” in clusters of genes or “Pan-cancer patterns of somatic copy number alterations” [3,17,37,38,39,40,41,42,43,44,56,78,84] are randomly selected from hundreds or thousands of other molecules that are found in multistep pathways in carcinogenesis. Isolation and identification of the numerous “molecular false-flags” that include EGFR, NFkB, PI3K, Kras, P53, BRAF, DPC4/SMAD4, BRCA2, ALK, IL-10, IL-12, TGF-β, c-Myc, BCR, ABL, CD11, CD22, require development of expensive and specific technologies. Such isolated molecules then become the foundation of costly drug development strategies and designs of clinical trials and claimed as ‘targeted’ therapies or “personalized” medicine. The average success rate for such claimed “targeted” therapies according to the admissions by government and industry is only 10%. That means that 90% (±5) of such expensive undertakings are worthless and failed to produce any benefit for cancer patients for over four decades [3,56,78]. A major biological reason for the repeatedly failed therapeutic approaches is that the politically-motivated decision makers (insiders or the establishment in the cancer community) of such expensive and out-of-focus projects have ignored or failed to consider the most basic biological principals of a living system which is the dynamics of compensatory, overlapping or duality/pleiotropy (bio-feedback) properties of immune and non-immune responses toward inhibition of specific components, particularly, in an already immune-compromised body of patients [3,38,39,40,56]. The designed drugs are primarily potent apoptotic agents/chemicals and/or antibodies that inhibit specific single or combination of growth factors that are selected in the disrupted, distorted, retarded and dysfunctional chaotic molecular environments in site-specific cancers [3,40,56]. An overall review of the toxicities of cancer claimed “targeted” therapies demonstrates severe additional disruptions in the biological, physical and mechanical integrities of multiple cellular components (e.g., mitochondrial oxidative metabolism, autophagy and lysosomal protein recycling pathways, extra-/intra-cellular and vascular membranes) that, in all likelihood, account for severe damages to the function of major organs/tissues (e.g., muscle, adipocytes, lymphoid organs, neuronal system and vasculature). The toxicities of such drugs often produce life-threatening side-effects such as cachexia, anorexia, sarcopenia, thromboembolism, drug-resistance and cancer relapse, leading to destruction of organ systems such as the kidneys, muscle, heart, lung and brain, or multiple organ failures (MOFs) and death [3,40,56]. The biological impact of cancer “targeted” therapies was recently compared with severe acute inflammatory diseases such as sepsis, meningitis, pneumonia or major trauma that are induced by potent pathogens (e.g., meningitis, salmonella, pneumococcal) that cause extensive expression of pro-apoptotic factors (“cytokine storm”, or “immune tsunami”) and aggressive disruption in the function of immune and non-immune systems leading to MOFs and death [3,40,56].

## 10. Questions, Major Knowledge Gaps and Future Research: Systematic Studies on Biology of Multistep Carcinogenesis toward Prevention and Therapy

Systematic understanding of the interactions between stimuli and host immune and non-immune response dynamics in tissues with similar or different resident (local) and recruited cell compositions seems to be logical projects for future investigations. In this regard, monitoring early changes of immune dynamics are perhaps the most important studies for potentially reversing and preventing the pathways toward carcinogenesis. The following is a proposed list of priorities, addressing major questions and knowledge gaps primarily based on the findings that we reported for models of acute and chronic inflammatory diseases in CALTs, majority of which could be applied and extended to other model systems:

(a) **Antigen clearing effects:** Understanding the basis for heterogeneities in immune response profiles (strong or weak acute inflammatory reactions) that were observed in acute phase in CALTs toward antigen challenges is important, since the extent of antigen permeability and access to inter-epithelial and sub-epithelial cells may produce significantly different outcomes. A strong type 1 reaction may restrict the penetration/exposure of antigen to trans-epithelial surface by an outward flow of fluids (e.g., mucus secretion and/or tearing) or copious leakage of vascular plasma from hyperpermeable vasculature. The increased tearing after a strong type 1 reaction also would wash away (dilute) or expel the antigen and protect the host during initial responses. However, increased or repeated exposures to stimuli and cumulative permeability of low levels of antigens may predispose the tissue toward antigen penetration. In contrast, a weak initial type 1 response, due to impaired function of MCs and/or B/plasma cells or GCs, may result in a greater net promotion of antigen penetration and/or increased epithelial exposure to higher doses of antigen or environmental toxins [3,37].
(b) **Role of mucus-secreting goblet cells (GCs):** Although little is known about the roles played by IgA-mucus-secreting GCs in the genesis of tumors, increased presence of these cells in the epithelial tissues of conjunctiva, and other epithelial tissues should be considered potentially important in acute inflammation, as well as, in the genesis and progression of chronic diseases or many cancers [3,37]. Involvement of GCs in diseases such as microglandular GCs, carcinoma or adenocarcinoid, crypt cell carcinoma, or mucinous carcinoid in tumors of the appendix, stomach and intestinal cancers, mucosal-associated diseases, colonic carcinomas, Barrett’s esophagus, conjunctival-associated lymphoma, lung cancer or neoplasia and tumors of endocrine systems have been documented [3,37,39,54,79,92]. In addition to the infiltration of eosinophils into GCs that we observed during the intermediate phase of inflammatory responses in CALTs [3,37,39], mature GCs tumors have been reported to be composed of infiltrating virus, or the glandular cords appeared to contain mucin-containing cuboidal cells with small eccentric nuclei [3,79].
(c) **Recruited macrophages:** Macrophages recruited into the CALTs during the chronic phase of immune responses, in all likelihoods, play crucial roles in their M2 phenotype (TAMs, wound healing or tumorigenic) to facilitate the induction of tumorigenesis. Synergistic expression of immune suppressive mediators such as histamine and/or PGE2 from “leaky” MCs combined with expression of suppressive mediators from TAMs into CALTs could further facilitate the growth properties of host tissue [3].

## 11. Oxidative Stress and Induction of Tumor-Associated MFs (TAMs) in Carcinogenesis

**Figure 7:**
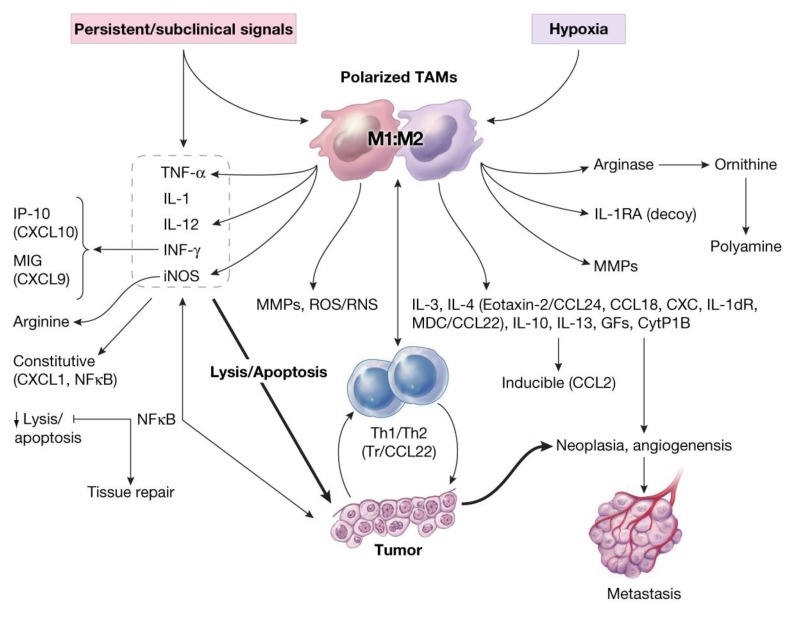
Schematic representation of oxidative stress-induced altered activities of macrophages (decreased M1/M2 ratios) during immune suppression and induction of tumorigenesis and angiogenesis in host. Reproduced from Khatami [39] with permission from Humana Press (Springer), all rights reserved.

Analyses of data from basic and clinical studies suggest that TAMs (activated M2 or alternative phenotype, tumorigenic), in contrast to classical M1 (resting MF phenotype, tumoricidal), express a number of cytokines/chemokines or decoy receptor molecules (e.g., dTNFR, IL-d1RA, dIL-1R, Eotaxin-2/CCL24, CCL18, CXC, IL-10, GFs, TGF-β, M-CSF, PGE2) that are immune suppressors and facilitate neoplasia, angiogenesis and tumor growth, and contribute to the epithelial-mesenchymal transition [3,15,20,22,39,42,47,50,57,85,92,93,94,95,96,97,98,99,100]. Expression of low level of chemokines such as CCL2 are suggested to contribute to the activation and transition of monocytes to macrophages, leading to expression of inducible-CCL2 during carcinogenesis and could serve as basis for localization and retention of macrophages in tumor microenvironment [57,93,96,97,98,99,100]. Figure 7, schematically represents the role of oxidative stress in the induction of alterations in the balance between tumoricidal and tumorigenic (Yin and Yang) properties of macrophages (M1: M2 or TAMs) in the direction of tumorigenesis [38,39]. 

M-CSF-1 and its receptor (M-CSF-1R) belonging to the cytokine family of c-fms proto-oncogene are implicated as the essential factors for normal monocyte development and trophoblastic implantation [20,22,57,93,94,95,96,97,98,99,100,101]. Binding of M-CSF to c-fms (a cell surface receptor of tyrosine kinase receptors family) results in dimerization and phosphorylation of c-fms and macrophage proliferation and support signal transduction capacity of TAMs in wound healing/growth-promoting pathways [57,93,94,95,96,97,98,99,100,101]. High expression of CSF-1, or c-fms and/or abnormal levels of M-CSF were also reported in chronic inflammatory conditions and ovarian adenocarcinoma, malignant germ cells of ovarian, lung, liver and other cancers at various stages of the disease [3,22,57,93,94,95,96,97,98,99,100,101].

## 12. Foundation of a Cancer Biomarkers Database: M-CSF as Prototype; Suitable Marker

As data on numerous claimed cancer biomarkers (CBs) accumulate in the literature, there is a growing need for developing effective strategies to identify and disseminate useful information for the oncology community to better serve the cancer-stricken public. Progress in developing worthy technologies and tools for cancer diagnosis has been extremely slow. In 1999, inflammatory mediators were introduced as potentially ideal targets (biomarkers) for early detection, chemoprevention and therapy of cancer [45]. In the last decade, a large body of cancer literature has focused on identification of numerous markers from site-specific cancers [3,44,45,57,65,92,93,94,95]. It is noteworthy that molecular analyses of traditional cancer biomarkers such as CA-125, CA-19-9, PSA, α-FP, CRP, or CEA, also reveal that nearly all of these markers are derived from immune/inflammatory responses [57].

In 2005, a first step toward the design of a foundation for a cancer biomarkers database tracking system was introduced by first standardizing cancer biomarkers criteria (data elements). Criteria for markers were identified and categorized from the review of available data on a wide range of clinical and basic research that represented prominent features of cancer markers [48]. The criteria selected for data elements included a number of known clinical features (e.g., diagnosis, surgical outcomes, epidemiologic data), origin or source of markers (e.g., blood, urine, saliva, tissue specimen), biological nature and molecular categories (e.g., proteins/peptides, lipids, hormones, metabolites, cytokines/chemokines, genetics/epigenetics, receptor molecules, enzymes, growth factors), comparison of specificity and sensitivity of markers with traditional markers (e.g., CA-125, CA-19-9, PSA, CEA) as well as, stages of technology development and/or validation status [48,57].

The tedious and time-consuming efforts in reviewing and categorizing prominent properties of a large number of markers became worthwhile for standardizing the criteria (data elements) on generic features of nearly all CBs (as a “passport” for marker identification) [48,57]. The next step involved introduction of a prototype for testing (tailoring) data elements. M-CSF was identified as a “Model” marker that would fit selected criteria for early detection of cancer. An ideal cancer biomarker for molecular diagnosis (e.g., M-CSF) was defined to possess special features such as:

(a) high specificity; marker will not be detected in healthy individual;
(b) high sensitivity; marker be detected at earliest stage of tissue growth or dysfunction (prior to cancer formation or when few cancerous cells are formed);
(c) marker possess predictive values; its levels in patient samples correlate with tumor stage;
(d) marker possess superior specificity and sensitivity compared with traditional markers;

Following determination of superior sensitivity and specificity of M-CSF over traditional markers (e.g., CA-125, CA-19-9), it was recommended that this cytokine was potentially a suitable marker that fulfilled important criteria for diagnosis of several cancers such as ovarian and pancreatic cancer; and potentially ready for validation and technology development (electronic devices) to be used in clinical settings [48,57].

## 13. Concluding Remarks: Promoting Immune Surveillance for Healthy Aging, Effective Cancer Prevention, Risk Assessment Formulation and Therapeutic Approaches

Given the crucial status of host tissue immune cell responses in the disease outcomes, systematic understanding of the host (resident) immune and non-immune interactions with those of recruited cells during oxidative stress are important when decisions on accurate risk assessment formulations and/or preventive and therapeutic approaches are strategized. Currently there are major biological, physical and mechanical knowledge gaps in our understanding of the heterogeneities and complexities of the interdependent interactions between cellular components in target tissues under a wide range of inflammatory conditions. Important knowledge gaps include the mechanisms that are involved in stimuli-induced generation, transmission and communication of danger signals within target tissues such as conjunctiva, lung airways, or gut, whose primary resident defense systems are mast cells, lymphoid tissues or mucus secreting goblet cells. Another biological puzzle is how specific signals that are transmitted from the activated resident cells in such tissues as CALTs or GALTs differ from other tissues with different resident cellular composition (e.g., liver, prostate, pancreas or breast). Little is also known about features of stimuli-induced expression of specific danger signals that are translated within the environment of host that would demand recruiting, infiltrating and interacting with other inflammatory cells (e.g., eosinophils or macrophages) and activated vasculature at various stages of tissue damage. In this regard, it is important to understand in details the nature of expression profiles of inflammatory cells that lead to extensive loss of architectural integrity and function in host tissues and the basis for tissue necrosis, in immune-privileged tissues and the induction of neurodegenerative and autoimmune diseases; or growth promotion, in immune-response tissues and the induction of site-specific cancers. Overall analyses of fragmented data on starvation-, or stimuli-induced alterations in the function of autophagy and protein clearance pathways including changes in the activities of lysosmal and Golgi apparatus, endoplasmic reticulum and mitochondrial oxidative metabolism, suggest contributions of these cellular defects in the outcomes of inflammatory diseases such as pancreatitis, neurodegenerative diseases, diabetes and cardiovascular complications and several cancers [26,80,102,103,104].

In summary, comprehensive understanding of the biology of immune surveillance is important not only for understanding the interrelationships between induction of acute and chronic inflammatory diseases and/or site-specific cancers, but also for helping to make informed decisions on promotion of the balance between tumoricidal (Yin) and tumorigenic (Yang) properties of immune system that would lead to effective prevention and therapeutic approaches and accurate assessment of risks toward improvement of public health.
